# A high throughput drug screening assay to identify compounds that promote oligodendrocyte differentiation using acutely dissociated and purified oligodendrocyte precursor cells

**DOI:** 10.1186/s13104-016-2220-2

**Published:** 2016-09-05

**Authors:** Karen D. Lariosa-Willingham, Elen S. Rosler, Jay S. Tung, Jason C. Dugas, Tassie L. Collins, Dmitri Leonoudakis

**Affiliations:** 1 Translational Medicine Center, Myelin Repair Foundation, Sunnyvale, CA 94085 USA; 2 Teva Pharmaceuticals, Biologics and CNS Discovery, Redwood City, CA 94063 USA; 3 Alios BioPharma, South San Francisco, CA 94080 USA; 4 Rigel Pharmaceuticals, South San Francisco, CA 94080 USA; 5 NGM Biopharmaceuticals, Inc., South San Francisco, CA 94080 USA

**Keywords:** Myelination, Oligodendrocyte, High throughput, Drug screening, Differentiation, Primary cell-based assay, Image analysis, Multiple sclerosis, Myelin basic protein

## Abstract

**Background:**

Multiple sclerosis is caused by an autoimmune response resulting in demyelination and neural degeneration. The adult central nervous system has the capacity to remyelinate axons in part through the generation of new oligodendrocytes (OLs). To identify clinical candidate compounds that may promote remyelination, we have developed a high throughput screening (HTS) assay to identify compounds that promote the differentiation of oligodendrocyte precursor cells (OPCs) into OLs.

**Results:**

Using acutely dissociated and purified rat OPCs coupled with immunofluorescent image quantification, we have developed an OL differentiation assay. We have validated this assay with a known promoter of differentiation, thyroid hormone, and subsequently used the assay to screen the NIH clinical collection library. We have identified twenty-seven hit compounds which were validated by dose response analysis and the generation of half maximal effective concentration (EC_50_) values allowed for the ranking of efficacy. The assay identified novel promoters of OL differentiation which we attribute to (1) the incorporation of an OL toxicity pre-screen to allow lowering the concentrations of toxic compounds and (2) the utilization of freshly purified, non-passaged OPCs. These features set our assay apart from other OL differentiation assays used for drug discovery efforts.

**Conclusions:**

This acute primary OL-based differentiation assay should be of use to those interested in screening large compound libraries for the identification of drugs for the treatment of MS and other demyelinating diseases.

**Electronic supplementary material:**

The online version of this article (doi:10.1186/s13104-016-2220-2) contains supplementary material, which is available to authorized users.

## Background

Multiple sclerosis (MS) is a devastating neurological disease, affecting over 2 million patients worldwide, caused by autoimmune-mediated destruction of the myelin sheaths that insulate and protect axons in the central nervous system. Most MS patients initially present with the relapsing-remitting form of MS (RRMS), in which cycles of immune-mediated axonal demyelination (relapse) are followed by periods of remyelination (remission). Over time, however, the majority of RRMS patients exhibit chronic, progressive decline in neurological function. This is believed to be the result of an accumulation of axonal damage, as well as an eventual loss of remyelination capacity [1]. Although current MS therapies for RRMS provide significant relief from relapse, none have yet been demonstrated to prevent disease progression and none have been effective in treating progressive forms of MS. All of the disease-modifying therapies approved for treatment of RRMS do so by targeting the pathologic immune response [2]. Currently, there are no therapies that function to directly protect or restore myelin, and identifying such therapies has been the focus of several groups in the field [3–7].

We have developed a high throughput drug screening assay to identify new therapeutic agents that accelerate the differentiation of immature OPCs into mature, myelin-producing OLs. The hope is that these newly differentiated OLs will accelerate the remyelination of axons following an immune attack. Here, we report the results of our screening campaign to identify drugs that stimulate oligodendrocyte differentiation. It is noteworthy that the assay that we employed differs in some details from those reported by others including (1) an OL toxicity prescreen which enabled lowering the concentrations of toxic compounds and (2) the utilization of freshly purified, non-passaged OPCs. We have screened the NIH collection of known drugs—a library of 727 drugs that have been approved for the treatment of various indications. Our screening efforts confirmed the activity of several drugs identified by other groups [3, 6, 7]. More importantly, we report the identification of a number of novel OL differentiation-promoting compounds not previously detected in other screening campaigns.

## Findings

### Development of an oligodendrocyte differentiation assay using acutely purified OPCs

**Fig. 1:**
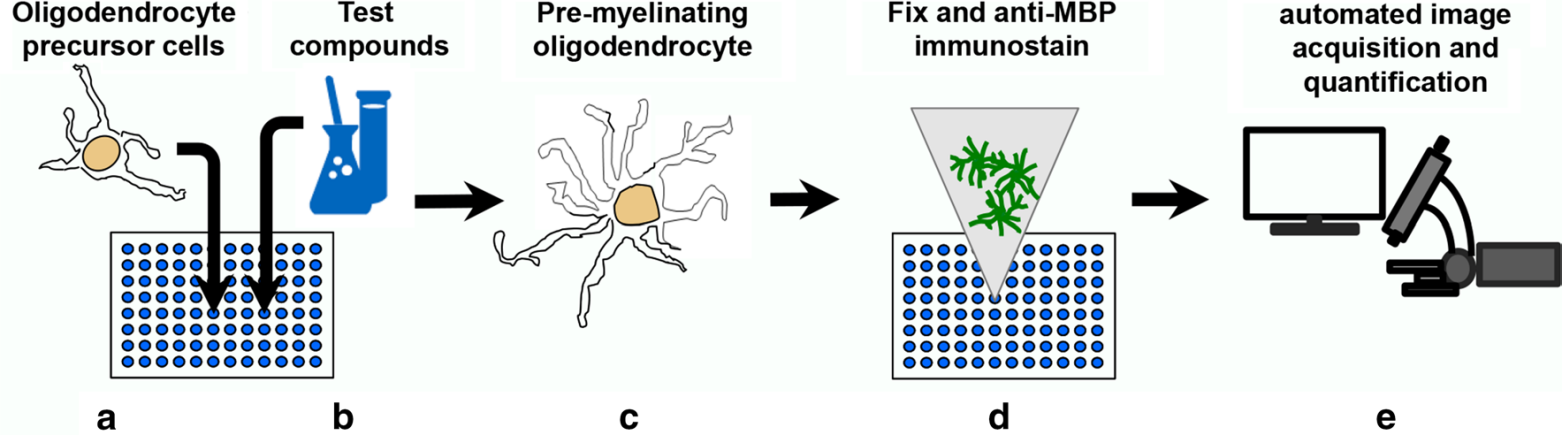
Flow scheme of the acute OL differentiation assay. **a** Purified oligodendrocyte precursor cells (OPCs) were cultured from P7 rat cortex and plated into PDL/laminin pre-coated 96-well plates. **b** One hour later test compounds were added. **c** OPC cultures with compound were maintained in culture for an additional 4 days. **d** On the 5th day with compound, cells were fixed and immunostained with anti-MBP antibodies and DAPI. **e** Images were acquired with automated fluorescence microscopy and scored phenotypically for OL differentiation (see “Methods” section)

**Fig. 2:**
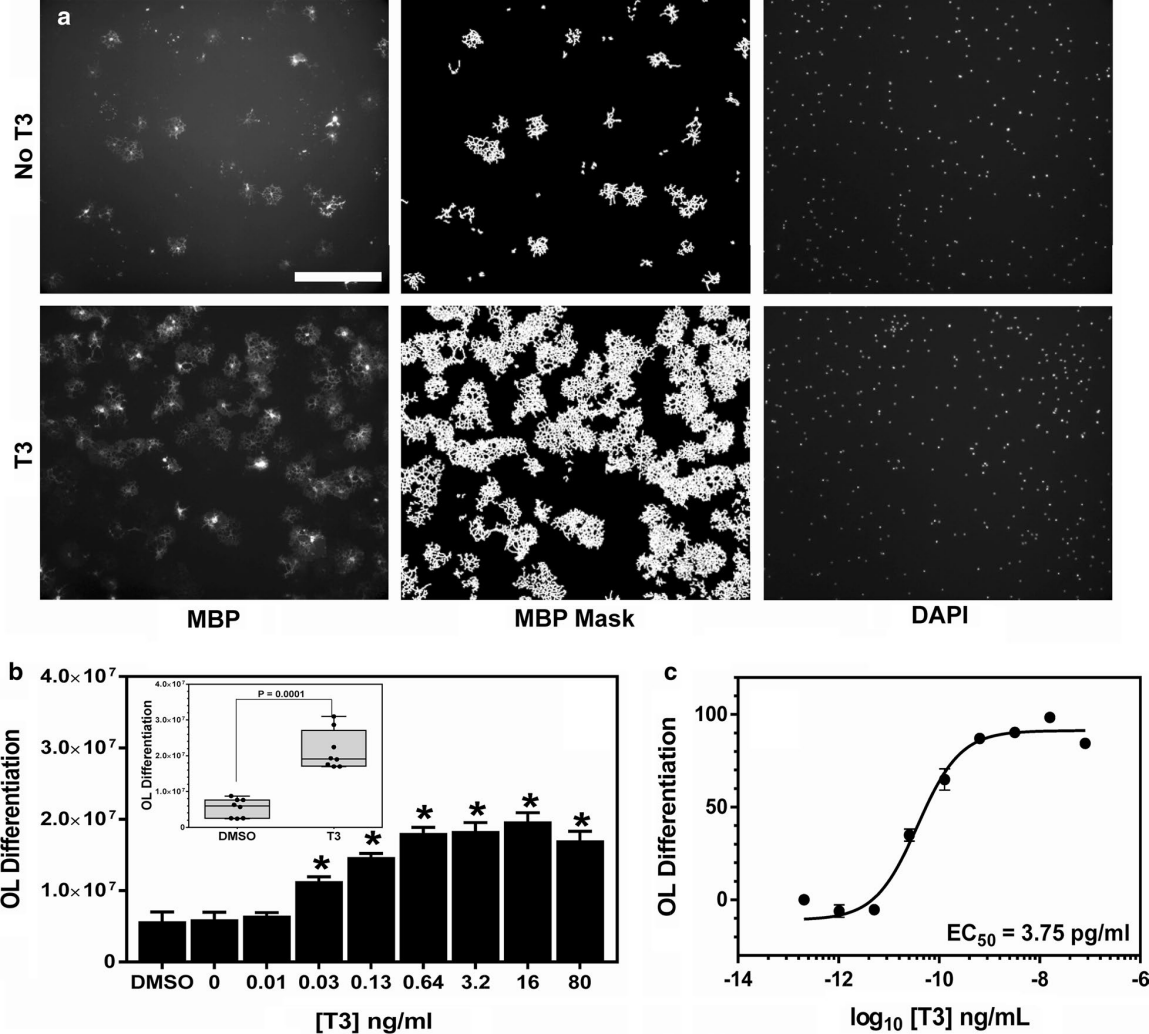
Acute OL differentiation assay quantification and validation. **a** *Left panels*, Full field images of acutely isolated and purified OPCs were differentiated for 4 days in the presence or absence of thyroid hormone (T3) and immunostained with anti-MBP antibodies. *Center panels*, digital masks were created using IN Cell software depicting the extent of threshold MBP staining of the images at *left*. *Right panels*, DAPI stained nuclei of the identical image filed at *left*. *Bar* = 200 µm. **b** The dose response activity of T3. *Asterisk* (*) denotes P values versus DMSO of <0.05, *t* test. *Inset*, the consistency and significance of T3 activity taken from eight independent plates. **c** The dose response curve and EC_50_ determination for T3 OL differentiation-promoting activity was calculated (16 fields per concentration, mean ± SEM)

In order to identify compounds with the potential to promote OL differentiation in vivo and thereby enhance remyelination, we first wanted to establish an in vitro assay system that would closely mimic conditions in vivo. To do this, we started by using OPCs acutely isolated from postnatal day 6–7 (P6–7) rat brains by O4 immunopanning [8]. This allowed us to obtain an enriched population of primary OPCs that could be used directly in cell culture assays without the need for extensive passaging and expansion in vitro. OPCs were plated directly onto a biological substrate (PDL/laminin) in 96-well view plates at moderate density to allow for sufficient room to expand processes during differentiation. We plated cells in serum-free medium lacking OPC mitogens (no PDGF or FGF) to allow a basal level of OL differentiation to commence, and as a positive control added thyroid hormone (T3, 40 ng/ml) to accelerate differentiation (Fig. 1, flow scheme) [9]. We found that T3 was able to consistently and significantly promote OL differentiation over the 0.1 % DMSO negative control, as measured by MBP expression after 4–5 days in culture in these conditions (Fig. 2a, b).

To quantify the extent of OL differentiation occurring in treatment conditions, we measured the overall amount of MBP expression/cell (see “Methods” section). Figure 2a depicts the mask created over MBP staining under which the MBP expression was quantified. The calculated MBP expression (OL differentiation) was then scaled to internal plate negative (0.1 % DMSO, set to 0) and positive (40 ng/ml T3, set to 100) control wells to generate percent OL differentiation score relative to the efficacy of positive control T3. This analysis of OL differentiation generated consistently significant results when T3 was compared to the DMSO negative control (Fig. 2b, inset). We used this analysis to determine the T3 dose response relationship and the EC_50_ value of 3.75 pg/ml (Fig. 2c).

### OL toxicity assessment of the NIH Clinical Collection (NCC) library of known drugs

Having established a consistent assay for measuring OL differentiation in vitro, the next step was to utilize this assay to assess the ability of a wide array of compounds to promote OL differentiation. Prior to library screening, we first established the maximum non-toxic dose of each compound in the library. Our rationale was that there may be a number of compounds that could be toxic to OPCs cultured in serum-free media when applied at high doses, but which may be beneficially active at lower doses. Consequently, these beneficial activities would be missed if compounds were only tested at high, toxic doses. Therefore, we added each compound in the NIH clinical collection (NCC) library to OPCs at 10, 1, and 0.1 µM concentrations. Three days later, we assessed viability of the treated cells by measuring AlamarBlue fluorescence, a measure of cell viability (see “Methods” section). We found that 17.7 % of the compounds tested were significantly toxic to cultured OPCs at 10 µM, but non-toxic at lower doses. An additional 9.5 % of tested compounds were significantly toxic even at lower tested doses (Additional file 1: Table S1). Appropriately, we established three sets of screening concentrations for library compounds: non-toxic compounds were tested at 10 and 2 µM, slightly toxic compounds (only toxic at 10 µM) were tested at 2 and 0.4 µM, and very toxic compounds were tested at 0.4 and 0.08 µM.

### NCC library screening for compounds that promote OL differentiation

Screening the NCC library of 727 known and FDA-approved drugs allowed us to test structurally distinct compounds with known safety profiles and biological activity in humans. Having established the peak non-toxic doses at which to test the NCC library compounds, we next screened this library using the acute OL differentiation assay. All compounds were tested at two concentrations, with positive (T3) and negative (0.1 % DMSO) control wells included on each assay plate. Additional file 2: Table S2 contains all of the acute OL differentiation assay NCC library screening data.

**Fig. 3:**
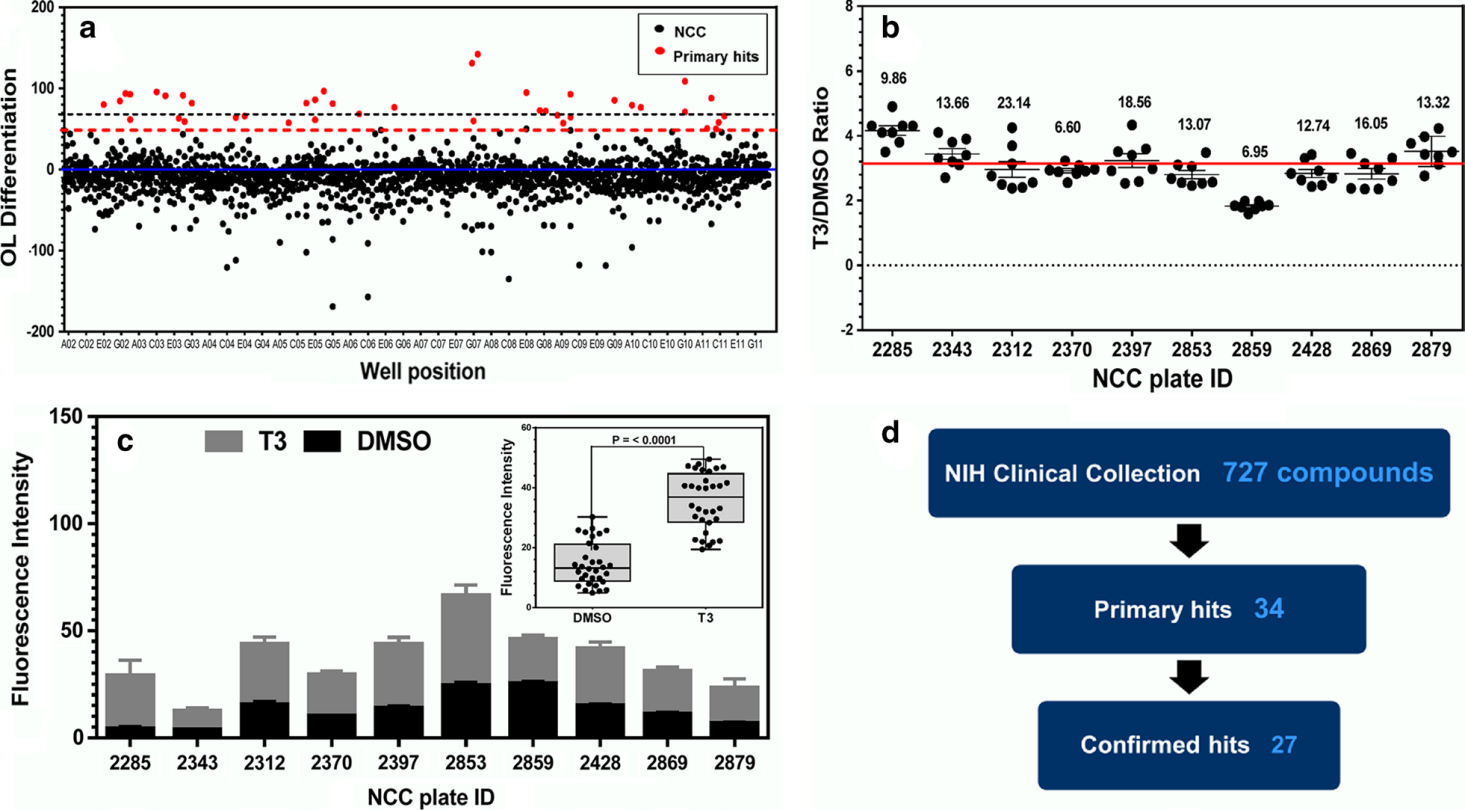
Analysis of the acute OL differentiation screen of the NCC compound library. **a** Dot plot representation of the entire high-throughput screening data set used to identify promoters of OL differentiation. Highlighted are the primary hit compound (*red*) activity. The mean response is indicated by the *blue solid line*. The *black dotted line* delineates the value of three standard deviations above the mean. The *red dotted line* delineates the ≥50 % positive control selection criteria. **b** Scatter plot representation of the high-throughput screening assay window. Using the T3/DMSO ratio as the window of measurable OL differentiation, the ratio of the positive (T3) to negative (DMSO) controls is depicted. The *red line* delineates the mean average ratio value for the entire NCC library screen = 3.1. The numbers above the scatter indicate the coefficient of variation (CV) for each plate. CV values <20 ± 5 were considered in the acceptable range. Each point is an average value from 16 fields. Each dot cluster represents eight control ratios from each NCC plate (n = 8, mean ± SEM). **c** Average raw values of DMSO and T3 for each library screening plate. *Inset*, The statistical analysis of the raw values of controls from all plates of the entire NCC library screen. **d** Summary of hit selection from the NCC library screen

Hits were classified as compounds that increased OL differentiation from baseline to at least 50 % of T3-promoted levels. To evaluate efficiency and validity of our screening method, we used a scatter plot to visualize compounds relative to three standard deviations (SD) from the mean (Fig. 3a). While not a criterion in our assay for hit selection, it provided a common statistical assurance that we were well out of false-positive hit rate (0.15 %) range. This was also confirmed visually when plotted alongside our ≥50 % T3 threshold (Fig. 3b). Positive control T3 versus DMSO values from the entire screen for OL differentiation were highly statistically significant per assay plate (Fig. 3b, c) and averaged over entire screen (Fig. 3c, inset) as well as robust (overall Z prime = 0.2), indicating an acceptable screen window and reproducibility. We identified 36 actives (5 % primary hit rate) able to promote OL differentiation at one or both concentrations tested (Fig. 3d; Additional file 2: Table S2). Two of these compounds were T3 and the T3 precursor thyroxine (T4), known promoters of OL differentiation, and thus not followed up in future studies.

### Independent confirmation and validation of identified hits

**Fig. 4:**
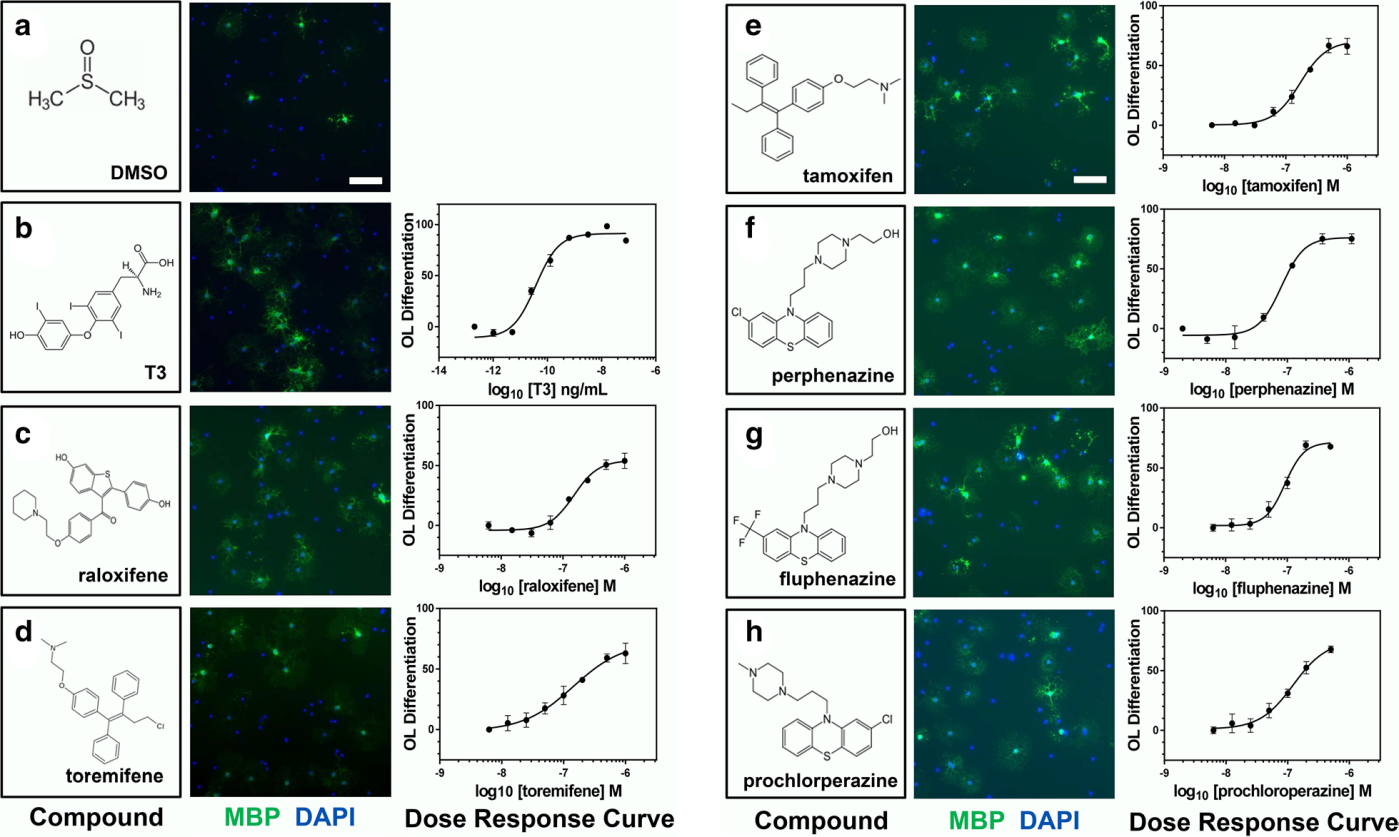
Structures, images, and EC_50_s of OL differentiation hits. **a**–**h** first column, Chemical structures and names of each hit compound and the controls, 0.1 % DMSO and T3. **a**–**h** second column, Example images of each hit compound directly from the library screening plate at the most efficacious concentration showing MBP (*green*) and DAPI (*blue*) staining. *Bar* = 200 µM. **a**–**h** third column, Representative OL differentiation dose–response curves used for computing the EC_50_ of each hit compound

**Fig. 5:**
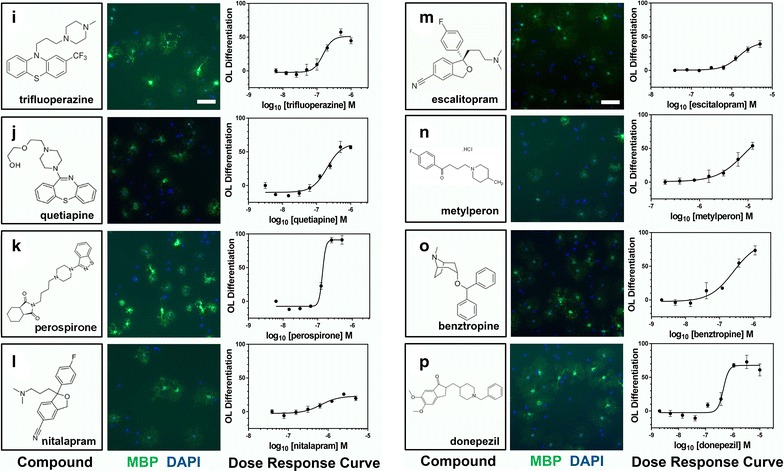
Structures, images, and EC_50_s of OL differentiation hits. **i**–**p** first column, Chemical structures and names of each hit compound and the controls, 0.1 % DMSO and T3. **i**–**p** second column, Example images of each hit compound directly from the library screening plate at the most efficacious concentration showing MBP (*green*) and DAPI (*blue*) staining. *Bar* = 200 µM. **i**–**p** third column, Representative OL differentiation dose–response curves used for computing the EC_50_ of each hit compound

**Fig. 6:**
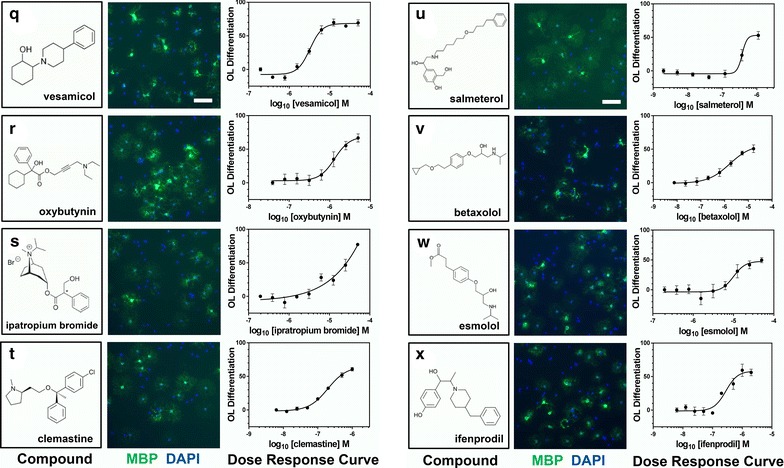
Structures, images, and EC_50_s of OL differentiation hits. **q**–**x** first column, Chemical structures and names of each hit compound and the controls, 0.1 % DMSO and T3. **q**–**x** second column, Example images of each hit compound directly from the library screening plate at the most efficacious concentration showing MBP (*green*) and DAPI (*blue*) staining. *Bar* = 200 µM. **q**–**x** third column, Representative OL differentiation dose–response curves used for computing the EC_50_ of each hit compound

**Fig. 7:**
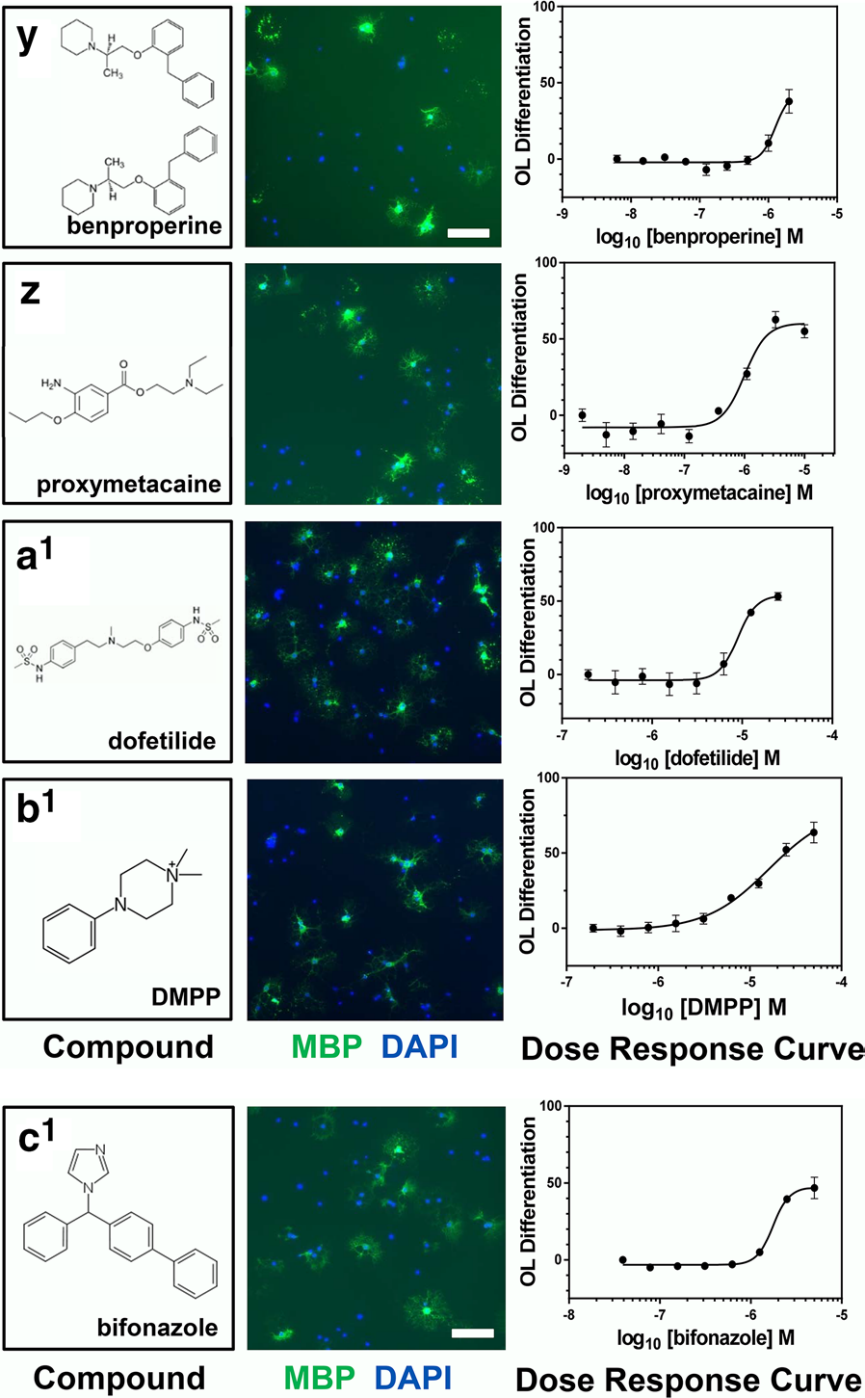
Structures, images, and EC_50_s of OL differentiation hits. **y**–**c** ^**1**^ first column, Chemical structures and names of each hit compound and the controls, 0.1 % DMSO and T3. **y**–**c** ^**1**^ second column, Example images of each hit compound directly from the library screening plate at the most efficacious concentration showing MBP (*green*) and DAPI (*blue*) staining. *Bar* = 200 µM. **y**–**c** ^**1**^ third column, Representative OL differentiation dose–response curves used for computing the EC_50_ of each hit compound

We next confirmed the differentiation-promoting activity of the 34 remaining identified compounds. We obtained all 34 hit compounds from various independent vendors to test in our OL differentiation assay. All compounds were tested at eight concentrations in at least two biological replicate experiments to generate dose-response curves for each candidate hit compound. Of the 34 initial hit compounds tested in this way, 26 (72 % hit confirmation rate) reproducibly promoted OL differentiation in a dose dependent manner (Table 1; Figs. 4, 5, 6, 7, 3d). Analysis of the EC_50_ values for each compound enabled the ranking of compound within each class. The average potency EC_50_, ranged from 30 nM to >20 µM (Table 1; Figs. 4, 5, 6, 7). Figures 4, 5, 6, 7 show each hit compound with the chemical structure, a representative MBP/DAPI screening image demonstrating numbers of OLs/field and OL morphology, and a representative OL differentiation EC_50_ curves. Table 1 shows the calculated average EC_50_ values for induction of differentiation obtained from independent biological replicates for the top hits from our initial OL differentiation screen. Based on the available literature on these previously characterized compounds, we grouped the hits by known mechanisms of action. Notably, our OL differentiation screen identified compounds from several distinct drug classes. Some of the hit compounds had been independently identified by other OL differentiation screening paradigms [3, 6, 7] (Table 2), whereas the refinement of using a toxicity prescreen and performing the assay on acutely purified OPCs allowed us to identify 16 additional novel compounds.

**Table 1:** Confirmed acute OL differentiation hits from the NCC library screen

Drug class	Compound	OL differentiation^a^	EC_50_, μM
SERM	Raloxifene	82	0.1
	Toremifene	96	0.1
	Tamoxifen	85	0.2
Tricyclic anti-depressants	Perphenazine	59	0.1
	Fluphenazine	91	0.1
	Prochlorperazine	80	0.1
	Trifluoperazine	60	0.2
	Quetiapine	109	0.4
Non-tricyclic anti-depressants	Perospirone	88	0.1
	Nitalapram	51	1.1
	Escitalopram	84	1.3
	Metylperon	64	7.3
Muscarinic	Benztropine	66	0.3
	Donepezil	142	0.5
	Vesamicol	91	1.4
	Oxybutynin	67	2.6
	Ipatropium	58	8.5
	Clemastine^b^	ND	0.2
Adrenergic	Salmeterol	52	0.4
	Betaxolol	86	4.2
	Esmolol	65	240
Ion-channel	Ifenprodil	93	0.4
	Benproperine	82	0.8
	Proxymetacaine	77	1.0
	Dofetilide	77	14
	DMPP	97	13
Anti-fungal	Bifonazole	66	1.7

*ND* no data. *DMPP* dimethylphenylpiperazinium

^a^Values indicate percent positive control

^b^Not part of the NCC library screen

**Table 2:** Technical comparison of acute OL differentiation assay with other OL differentiation assays

Technical details	MRF-TMC^a^	Deshmukh [3]	Mei [6]	Najm [7]
Pre-toxicity screen	●			
Rat OPCs	●	●	●	
Mouse OPCs			●	●
Acutely-derived	●			
Passaged/expanded OPCs		●	●	
iPSC-derived OPCs				●
Cortex-derived OPCs	●		●	
Optic nerve-derived OPCs		●		
MBP/PDGFRα/micropillar wrapping analysis			●	
MBP expression analysis	●	●		●

^a^Myelin Repair Foundation, Translation Medicine Center

Although we observed that all actives could promote not only MBP expression, but also a morphological shift typical to OL differentiation (Figs. 4a–h, 5 i–p, 6q–x, 7y–c^1^), we nonetheless confirmed that active compounds could promote other markers of OL differentiation. In addition to MBP, we tested the ability of the 27 confirmed hit compounds to promote expression of 2′, 3′-cyclic-nucleotide 3′-phosphodiesterase (CNPase) and Galactocerebroside (GalC), two additional established markers of OL differentiation [10, 11]. All 27 confirmed hit compounds promoted CNP and GalC in our acute OL differentiation assay (Additional file 1: Figure S1A, B is shown).

## Discussion

### Development of an acute OL differentiation compound screening assay

In an effort to identify possible therapeutic candidate compounds for multiple sclerosis and other demyelinating diseases, we have developed novel oligodendrocyte assays amenable to high throughput screening. We have established the first OL differentiation assay to utilize freshly purified, unpassaged OPCs. This distinguishes our assay from other OL differentiation assays used for drug discovery efforts, which used highly passaged OPCs or iPS-derived OPCs. Additionally, we used an OL toxicity pre-screening assay to customize our library screening efforts to account for compounds that may be toxic at high concentration, but efficacious at lower concentrations. This is an unusual precaution to take, as it is standard practice to screen an entire library at a single concentration (e.g., 10 µM) to minimize labor and time. Such a library is thus very different than a typical HTS library composed of novel, unknown and un-optimized compounds. This approach proved fruitful, as we identified several OL differentiation-promoting drugs in the NCC library that were not identified in another screen [7] that used this same library. Below is a discussion of some of the top hit compounds identified from our OL differentiation assay and their possible relevance to multiple sclerosis.

Other in vitro OL differentiation assays used for compound screening have only assessed differentiation using purified OPCs adapted to culture conditions by multiple passages [3], or differentiated from induced pluripotent stem cells [7]. Mei et al. developed an HTS assay incorporating OL differentiation in the presence of inert micropillars allowing the quantification of pillar wrapping as a surrogate for myelination [6]. We have noted the technical similarities and differences of our OL differentiation assay with these other studies in Table 2. The lead compounds that overlap with our findings are summarized in Table 3 and represent only about half of the leads we identified (in particular, the tricyclic anti-depressants and muscarinic drug classes), with the remaining compounds being identified and reported for the first time in our screen.

**Table 3:** Comparison of acute OL differentiation hits in the NCC library with hits reported in other OL differentiation assays

Confirmed NCC library hits		OL differentiation assays			
Drug class	Compound	MRF-TMC^a^	Deshmukh [3]	Mei [6]	Najm [7]
SERM	Raloxifene	●			
	Toremifene	●			
	Tamoxifen	●			●
Tricyclic anti-depressants	Perphenazine	●	●		
	Fluphenazine	●	●		
	Prochlorperazine	●			
	Trifluoperazine	●	●		
	Quetiapine	●		●	●
Non-tricyclic anti-depressants	Perospirone	●			
	Nitalapram	●			
	Escitalopram	●			
	Metylperon	●			
Muscarinic	Benztropine	●	●	●	●
	Donepezil	●			
	Vesamicol	●			●
	Oxybutynin	●	●	●	
	Ipatropium	●	●	●	
	Clemastine^b^	●	●	●	
Adrenergic	Salmeterol	●	●		
	Betaxolol	●			
	Esmolol	●			
Ion-channel	Ifenprodil	●			
	Benproperine	●			
	Proxymetacaine	●			
	Dofetilide	●			
	DMPP	●			
Anti-fungal	Bifonazole	●			

^a^Myelin Repair Foundation, Translation Medicine Center

^b^Compound not part of the NCC library screen

It should be noted that this in vitro OL differentiation screening assay only identifies compounds that promote OL differentiation and does not address whether the differentiated OLs also promote myelination. Additionally, this assay is performed in purified monoculture absent of other cell types that influence OL differentiation and myelination, e.g. neurons and astrocytes. Furthermore, this assay is performed under ideal OL differentiation conditions, unlike the inflammatory conditions present demyelination lesions. One should also be aware that the compounds identified in this screen have only been tested on rat OPCs. Mei et al. demonstrated cross-species compound activity in their OL differentiation assay, however, compounds identified from our OL differentiation screen have not been verified on mouse or human cells. Testing these compounds on OLs derived from human iPSCs would greatly increase the potential therapeutic value of these compounds. Nevertheless, OL differentiation-promoting compounds have the potential to increase the number of myelination competent OLs and therefore facilitate myelination. This hypothesis has been supported by the previously published work mentioned above that have identified pro-OL differentiating compounds that have facilitated myelination in vivo [3, 6, 7].

### Confirmed hits group into multiple mechanistic classes

**Fig. 8:**
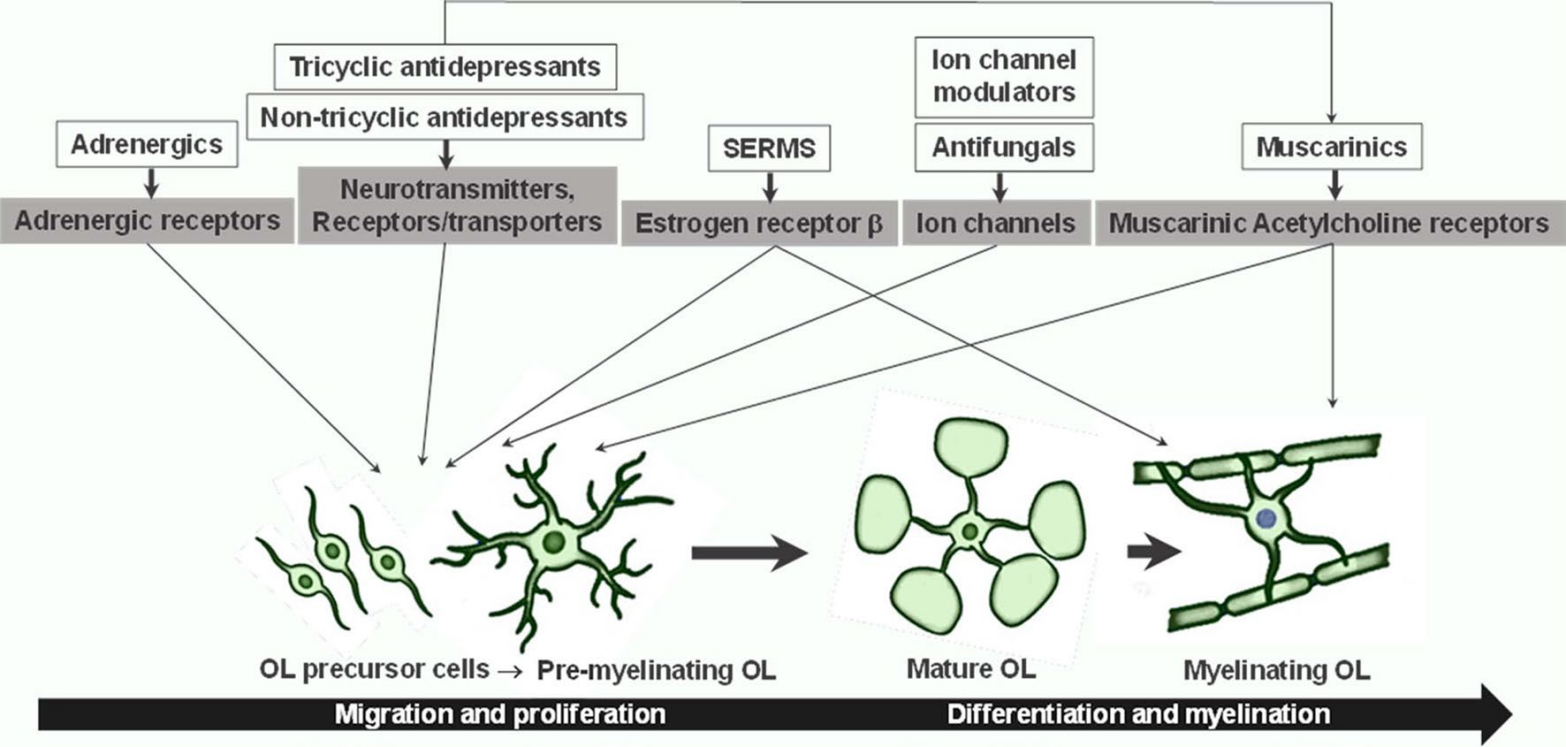
Possible hit compound signaling pathways involved in OL differentiation and myelination. The 27 compounds identified from the acute OL differentiation screen were grouped into seven classes: muscarinic receptor antagonists, andrenergic agonists, tricyclic antidepressants, non-tricyclic antidepressants, selective estrogen receptor modulators (SERMs), ion channel modulators and antifungals. Based on a literature review, these compound classes were further categorized into the most likely steps in OL development where compounds may be active. Note that some of the tricyclic antidepressents and non-tricyclic antidepressents have off-target muscarinic activity. Also note that the OL differentiation-promoting mechanisms of these compounds may not necessarily act through the same mechanisms as the known FDA-approved indication target

We found that the hits identified in the OL differentiation assay (Table 1) could be assigned into six broad categories, falling into common functional or structural categories. We have grouped these compounds into possible signaling pathways that feed into OL differentiation (Fig. 8).

Benztropine, donepezil, oxybutynin, vesamicol, and ipratropium all perturb the muscarinic acetylcholine signaling pathway. Indeed, the muscarinic receptor pathway has been previously implicated in OL differentiation and benztropine, oxybutynin and ipratropium were identified in other OL differentiation screens [3, 6, 7] (see Table 2, comparison). To supplement this class, we also tested clemastine, another muscarinic receptor antagonist characterized by Mei et al. [6] and found it too could promote OL differentiation in our assay similar to benztropine (Table 1; Figs. 4, 5, 6, 7; Additional file 1: Figure S1A). Both benztropine and clemastine have shown efficacy in stimulating remyelination in in vivo models [3, 6]. Previous work has demonstrated that M2 muscarinic receptor agonism has mitogenic activity increasing PDGFR∝ expression to keep OPCs in a proliferative state [12]. Further, it was recently shown that treatment with solifenacin, an FDA-approved M3 muscarinic receptor antagonist, promoted oligodendrocyte differentiation of transplanted hOPCs in hypomyelinated shiverer/rag2 brain [13].

A second compound class uniquely identified by our OL differentiation assay was the selective estrogen receptor modulators (SERMs): raloxifene, toremifene, and tamoxifen. These compounds may be acting through the estrogen β receptor (EβR) and the PI3 K/Akt/mTOR signaling pathway, previously shown to stimulate OL differentiation and remyelination in vivo in demyelinating animal models of MS [14–18].

A third compound class includes perphenazine, prochlorperazine, fluphenazine, trifluorperazine, and QTP: five tricyclic antidepressant molecules with structural similarity believed to act primarily as serotonin/norepinephrine reuptake inhibitors (SNRIs) by blocking the serotonin transporter (SERT) and the norepinephrine transporter (NET). It must also be noted that these compounds also have significant off-target activity, including muscarinic antagonistic activity among others, which may account for OL differentiation-promoting activity. Perospirone, escitalopram, citalopram, metylperon, and bupropion are loosely grouped together as a class of non-tricyclic antidepressants that, with the exception of escitalopram and citalopram, do not share much structural similarity. These compounds have the properties of being selective serotonin reuptake inhibitors (SSRIs—escitalopram, citalopram, bupropion) or dopamine receptor antagonists (perospirone, metylperon). Since OPCs/pre-myelinating OLs express SERTs, NETs, serotonin receptors, and dopamine receptors [19], it is possible that the antidepressant class of compounds may function by modulating neurotransmitter levels and/or neurotransmitter receptor activity to facilitate OL differentiation [20].

Salmeterol, betaxolol, and esmolol all interact with the adrenergic receptor (AR) pathway [21]. There is a paucity of data on AR function in OL differentiation. ARs are expressed in OL precursors [19] and the ∝1-AR has been demonstrated to play a role in OL specification and differentiation in vivo [22].

Finally, a broad class of ion channel modulators was identified: ifenprodil, benproperine, proxymetacaine, dofetilide, and dimethylphenylpiperazinium (DMPP). DMPP acts through the stimulation of the nicotinic acetylcholine receptor (nAChR) which may play a role in OL differentiation. Interestingly, it was recently demonstrated that donepezil-induced OL differentiation was mediated by the nACHR. Ifenprodil is an NMDA receptor (NMDAR) antagonist. It has been shown that NMDARs are present in precursor, immature and mature OLs [23, 24]. One additional active compound, bifonazole, is a known antifungal agent. A number of azole-containing anti-fungals have known effects on ion channels [25], but it is not known what role these may have in OL differentiation. By interacting with ion channels, these compounds may alter intracellular ion levels within OPCs/pre-myelinating OLs to facilitate OL differentiation [26].

### Relevance to multiple sclerosis

There are many examples where repurposed drugs are making an impact in the field of MS. A few of note are BG-12 (Tecfidera®), a modification of a psoriasis drug; fingolimod (Gilenya®), originally developed for transplantation and alemtuzamab (Lemtrada™), originally an oncology drug. We found that a fair number of our hits aligned with current MS repositioning efforts in terms of patents, preclinical work, and clinical trials and are briefly cited here.

The SERMs represent a promising new class of compounds that promote OL differentiation not identified in other screens. Modulation of the estrogen receptor has been demonstrated to be effective in preventing demyelination in vivo and to stimulate differentiation and remyelination [27–30]. Specifically, raloxifene (Evista®) was shown to be effective in suppressing EAE [31]. The pregnancy hormone estriol (Trimesta™) has shown efficacy in a clinical trial of MS reducing the lesion numbers and volumes [32].

A number of hits that we identified are covered in patents for oligodendrocyte directed differentiation. Notably trifluoperazine (Stelatzine®), benztropine (Cogentin®), and ipatropium (Atrovent®) are patented for the use of neurotransmitter receptor modulating agents for inducing OL differentiation, as well as methods for treating subjects with such agents in a demyelinating disease.

Other hits have been evaluated or will soon be tested in human MS trials. QTP (Seroquel XR®), an antipsychotic approved for schizophrenia is currently in clinical trials to determine tolerability to people with relapsing remitting multiple sclerosis (RRMS) and progressive MS (Clinicaltrials.gov identifier: NCT02087631). Selective serotonin re-uptake inhibitors (SSRIs) such as nitalapram (Celexa®) and escitalopram (Lexapro®) have also been in human MS trials. Nitalapram is one of three other SSRIs tested as an add-on with fingolimod, a current immunomodulatory treatment for MS (REGAIN Trial NCT01436643) in RRMS patients with depression. Escitalopram has been in a study to test improvement of depression in patients of MS or ALS (Trial NCT00965497). Donepezil (Aricept®), used as a treatment for dementia, has been used in a study to improve cognitive dysfunction in MS patients (NCT00315367) [33–35]. Oxybutynin (Ditropan XL®**)** is used currently in symptom management for MS to treat overactive bladder in MS (SONIC trial NCT00629642). Clemastine fumarate (Tavist®) originally approved as a first-generation antihistamine, is active in a current study to assess the drug as a remyelinating agent is RRMS patients (ReBUILD trial NCT02040298). Though not a hit in our screen, albuterol (compound #4, Additional file 3: Table S2), a short acting β2 adrenergic receptor agonist, was tested in a clinical trial (Trial NCT00039988) as an add-on therapy to capaxone (glatiramir acetate) for patients with RRMS and showed improved clinical outcomes. Perhaps the longer acting β2 adrenergic receptor agonist, salmeterol, which we identified in our screen, may also provide some clinical efficacy.

To our knowledge, the remainder of our confirmed hits has not been tested in any demyelinating diseases.

## Conclusion

We have demonstrated here an OL differentiation assay with the robustness, scale and reproducibility amenable to HTS drug discovery. The assay was designed to preserve primary cell, in vivo-like characteristics in culture by using only acutely isolated cells (OL differentiation assay) and limited subculture to a single expansion (OL toxicity assay). Thus, we avoided undesirable changes in cellular phenotype (e.g. gene expression and/or unstable karyotypes) that multiple cell passages or derivation from large-scale stem cell cultures may introduce [3, 7, 36, 37]. Additionally, the OL toxicity pre-screen allowed us to reduce the top concentrations of toxic compounds and was essential to identifying compounds that were more efficacious at lower concentrations. Future studies should focus on testing these novel OL differentiation-promoting compounds both in vitro with human cells as well as in vivo models of MS. Furthermore, this assay is well-suited for screening larger compound libraries with novel chemical matter.

## Methods

### Reagents

High glucose Dulbecco’s modified eagle medium (DMEM), neurobasal medium (NB), Earle’s balanced salt solution (EBSS), l-glutamine, fetal bovine serum (FBS), penicillin/streptomycin, sodium pyruvate, diamidino-2-phenylindole, dilactate (DAPI) were purchased from life technologies (Carlsbad, CA, USA). Normal goat serum, forskolin, triiodothyronine (T3, thyroid hormone), vitamin B12, hydrocortisone, d-biotin, apotransferrin, putrescine, progesterone, sodium selenite, poly-d-lysine (PDL), recombinant human (rh) insulin, bovine serum albumin, *N*-acetyl-l-cysteine, sodium phosphate (mono- and dibasic), sodium chloride, tris base, l-lysine, sodium azide, tunicamycin and DMSO were obtained from Sigma-Aldrich (St. Louis, MO, USA). Trace elements B, Dulbecco’s phosphate buffer saline (dPBS) and trypsin 0.05 %-EDTA were purchased from Mediatech, Inc. (Manassas, VA, USA). Human ceruloplasmin was purchased from EMD Millipore (Billerica, MA, USA). Recombinant rat (rr) CNTF, rrIFNγ, rrTNFα, rhNT-3 and rhPDGF-AA were purchased from PeproTech (Rock Hill, NJ, USA). Laminin was obtained from Trevigen (Gaithersburg, MD, USA). DNase, papain and ovomucoid inhibitor were purchased from Worthington Biochemical Corporation (Lakewood, NJ, USA). Paraformaldehyde solution was purchased from Electron Microscopy Science (Hatfield, PA). alamarBlue® (AB) was purchase from AbD Serotech (Killington, UK). Packard 96-well Viewplates were purchased from Perkin Elmer (Waltham, MA, USA). Falcon TC 96-well plates were purchase from corning (Corning, NY). Additional file 4: Table S3 lists the primary antibodies and their dilutions used in this study.

### Compounds

All compounds in the NIH Clinical Collection (NCC) library were supplied in DMSO at 10 mM in 96-well plates. Hit compounds were purchased as powders and stock solutions were dissolved in DMSO to 10 mM (see Additional file 2: Table S1, Additional file 3: Table S2 for complete listing of compounds).

### Isolation and culture of neonatal rat OPCs

All animal work was carried out in strict accordance with the recommendations in the Guide for the Care and Use of Laboratory Animals of the National Institutes of Health. The protocol was approved by the Institutional Use and Care and Use Committee at the Molecular Medicine Research Institute (Sunnyvale, CA). Animals used for OPC isolation in these studies were euthanized by decapitation. OPCs from brains of P6-P7 Sprague–Dawley rat pups (Charles River, Wilmington, MA, USA) were enriched by immunopanning as previously described (Dugas et al. 2006). Briefly, single cell suspensions were obtained from papain-digested neonatal brains and depleted of mature glial cells by successive panning with RAN-2- and GalC-coated plates, followed by enrichment for OPCs using O4-coated plates. OPCs were plated in differentiation media (DMEM, 100 U/ml Penicillin/100 µg/ml streptomycin, 2 mM l-glutamine, 1 mM Na pyruvate, 5 µg/ml insulin, 5 µg/ml *N*-acetyl-l-cysteine, 1× trace elements B,10 ng/ml d-Biotin, 100 µg/ml BSA, 100 µg/ml apo-transferrin, 16 µg/ml putrescine, 60 ng/ml progesterone, 40 ng/ml sodium selenite 5 ng/ml forskolin, 10 ng/ml CNTF, 1 ng/ml NT-3).

### Oligodendrocyte toxicity assay

OPCs were expanded in vitro by seeding into PDL-coated tissue culture flasks at 1000–2000 cells/cm^2^ in differentiation media supplemented with 10 ng/ml PDGF-AA. Proliferating OPCs were fed every 2–3 days and supplemented with 2–3× concentration of PDGF as needed to prevent OPC differentiation. After 6–7 days of proliferation, OPCs were harvested by trypsinization, plated into PDL-coated 96-well Falcon plates and centrifuged at 200×*g* to facilitate cell attachment, survival, and even distribution of cells. Expanded OPCs were seeded in differentiation media at 10,000 cells/well and incubated for 2 h at 37 °C, 10 % CO_2_ prior to addition of compounds. The NCC library was diluted and added to cells at 10, 1, and 0.1 µM; each concentration was tested in quadruplicate. As a positive control for the induction of toxicity, 5 ng/ml tunicamycin was added to 8 wells in each plate. Three days later, a 1:10 dilution of alamarBlue^®^ (AB) was added to each well and cells were incubated 4 h at 37 °C, 10 % CO_2_. AB fluorescence was measured using a PerkinElmer Victor II fluorescence microplate reader (PerkinElmer, Waltham, MA) with 560 nm excitation/590 nm emission filters. For library screening, the highest concentration of each compound was adjusted to a minimally toxic concentration based on this toxicity data. Compounds were additionally tested at a second concentration that was 1/5th of the top concentration. The final concentration of DMSO in each well was 0.1 %.

### Acute oligodendrocyte differentiation assay

Acutely enriched OPCs were plated in differentiation media at 5000 cells/well into PDL/laminin coated 96-well view plates and centrifuged at 200×*g* to facilitate cell attachment, survival, and even distribution of OPCs. OPCs were pre-incubated for 1–2 h at 37 °C in 10 % CO_2_, followed by the addition of test compounds in quadruplicate. Controls were added in eight replicate wells, negative control = 0.1 % DMSO; positive control = 40 ng/ml T3. The day of OPC plating was considered DIV0. On DIV4, cells were fixed, immunostained for myelin basic protein (MBP) expression, imaged and quantified as described below.

### Immunofluorescence staining and imaging

Following compound treatment, media was removed leaving 50 μl/well using an ELx405 microplate washer (BioTek, Winooski, VT, USA), the automated plate washer was also used for all subsequent washing steps during the staining. Cells were fixed for 14 min with 4 % paraformaldehyde at room temperature (RT). Following fixation, plates were washed with 1 ml PBS leaving 50 μl/well. Cells were then incubated in blocking buffer (10 % normal goat serum (NGS) diluted in antibody buffer: 150 mM NaCl, 50 mM Tris Base, 1 % BSA, 100 mM l-lysine, 0.04 % sodium azide, pH 7.4), with 0.4 % Triton X-100 for 1 h at RT, then stained overnight at 4 °C with rat anti-MBP antibodies (Additional file 4: Table S3) diluted in blocking buffer with 0.08 % Triton X-100. The cells were washed and incubated with secondary antibodies and 0.3 μM DAPI for 1 h at RT. After a final wash, 100 μl of PBS was added to each well and plates imaged. Images were captured with a Nikon Eclipse TE-2000-U microscope, Zyla cMOS megapixel camera (ANDOR Technology, Belfast, UK), fitted with an automated stage controlled by NIS Elements AR software 4.0 (Melville, NY, USA). An air 10× lens was used to capture 2–4 images per well with 16 bit resolution, 2560 × 2160 pixels. Images for each assay run were captured using identical camera settings. Images were exported as TIFF files for analysis and quantification.

### Image quantification

TIFF files were analyzed using custom algorithms created with IN Cell Investigator Developer Toolbox (GE Health Sciences, Piscataway, NJ, USA). For each well of OL differentiation assay, four images were analyzed per well and the data from the duplicate wells was combined and averaged (total of eight images per test condition). For cytokine protection assay, two images were analyzed per well and the data from triplicate wells was combined and averaged (total of six images per test condition). The overall amount of MBP expression/cell was quantified in each image by calculating the density × area of MBP staining, and normalizing to the total number of DAPI-stained nuclei in the same field. The calculated extent of MBP expression (OL differentiation) was then scaled to internal plate negative (0.1 % DMSO) and positive controls (40 ng/ml T3) to generate percent OL differentiation scores relative to the efficacy of positive control. For the cytokine protection assay, the positive controls were either no insult or 1.1 μM QTP. This analysis consistently generated quantitatively similar results when scaled to internal plate negative and positive control wells.

### Relative EC_50_ analysis

EC_50_ values were obtained by fitting the data to a sigmoidal dose–response curve-fitting function (Prism, GraphPad, San Diego, CA, USA). Serial dilutions of eight to ten different concentrations with three or four data points per concentration were used for curve fitting. Experiments were repeated at least two times.

### Statistical methods

For all experiments, assuming normal distribution, two-tailed *t*-tests were used to evaluate comparisons between two groups.

## Additional files

10.1186/s13104-016-2220-2 Confirmation of OL differentiation hit compounds with multiple markers.
10.1186/s13104-016-2220-2 NCC library toxicity screen data set.
10.1186/s13104-016-2220-2 NCC library acute OL differentiation screen data set.
10.1186/s13104-016-2220-2 Antibodies.

## Abbreviations

CNS: central nervous system
MS: multiple sclerosis
OL: oligodendrocytes
OPCs: oligodendrocyte precursor cells
DIV: days in vitro
MBP: myelin basic protein
DMSO: dimethylsulfoxide
HTS: high throughput screening
NCC: NIH clinical collection

## References

[1] Franklin RJ, Edgar JM, Smith KJ, ffrench-Constant C (2012). Neuroprotection and repair in multiple sclerosis. Nat Rev Neurol.

[2] Ransohoff RM, Hafler DA, Lucchinetti CF (2015). Multiple sclerosis-a quiet revolution. Nat Rev Neurol.

[3] Deshmukh VA, Tardif V, Lyssiotis CA, Green CC, Kerman B, Kim HJ, Padmanabhan K, Swoboda JG, Ahmad I, Kondo T (2013). A regenerative approach to the treatment of multiple sclerosis. Nature.

[4] Jepson S, Vought B, Gross CH, Gan L, Austen D, Frantz JD, Zwahlen J, Lowe D, Markland W, Krauss R (2012). LINGO-1, a transmembrane signaling protein, inhibits oligodendrocyte differentiation and myelination through intercellular self-interactions. J Biol Chem.

[5] Lee S, Leach MK, Redmond SA, Chong SY, Mellon SH, Tuck SJ, Feng ZQ, Corey JM, Chan JR (2012). A culture system to study oligodendrocyte myelination processes using engineered nanofibers. Nat Methods.

[6] Mei F, Fancy SP, Shen YA, Niu J, Zhao C, Presley B, Miao E, Lee S, Mayoral SR, Redmond SA (2014). Micropillar arrays as a high-throughput screening platform for therapeutics in multiple sclerosis. Nat Med.

[7] Najm FJ, Madhavan M, Zaremba A, Shick E, Karl RT, Factor DC, Miller TE, Nevin ZS, Kantor C, Sargent A (2015). Drug-based modulation of endogenous stem cells promotes functional remyelination in vivo. Nature.

[8] Dugas JC, Tai YC, Speed TP, Ngai J, Barres BA (2006). Functional genomic analysis of oligodendrocyte differentiation. J Neurosci.

[9] Barres BA, Lazar MA, Raff MC (1994). A novel role for thyroid hormone, glucocorticoids and retinoic acid in timing oligodendrocyte development. Development.

[10] Raff MC, Mirsky R, Fields KL, Lisak RP, Dorfman SH, Silberberg DH, Gregson NA, Leibowitz S, Kennedy MC (1978). Galactocerebroside is a specific cell-surface antigenic marker for oligodendrocytes in culture. Nature.

[11] Sprinkle TJ (1989). 2′,3′-cyclic nucleotide 3′-phosphodiesterase, an oligodendrocyte-Schwann cell and myelin-associated enzyme of the nervous system. Crit Rev Neurobiol.

[12] De Angelis F, Bernardo A, Magnaghi V, Minghetti L, Tata AM (2012). Muscarinic receptor subtypes as potential targets to modulate oligodendrocyte progenitor survival, proliferation, and differentiation. Dev Neurobiol.

[13] Abiraman K, Pol SU, O’Bara MA, Chen GD, Khaku ZM, Wang J, Thorn D, Vedia BH, Ekwegbalu EC, Li JX (2015). Anti-muscarinic adjunct therapy accelerates functional human oligodendrocyte repair. J Neurosci.

[14] Acs P, Kipp M, Norkute A, Johann S, Clarner T, Braun A, Berente Z, Komoly S, Beyer C (2009). 17beta-estradiol and progesterone prevent cuprizone provoked demyelination of corpus callosum in male mice. Glia.

[15] Khalaj AJ, Yoon J, Nakai J, Winchester Z, Moore SM, Yoo T, Martinez-Torres L, Kumar S, Itoh N, Tiwari-Woodruff SK (2013). Estrogen receptor (ER) beta expression in oligodendrocytes is required for attenuation of clinical disease by an ERbeta ligand. Proc Natl Acad Sci USA.

[16] Kumar S, Patel R, Moore S, Crawford DK, Suwanna N, Mangiardi M, Tiwari-Woodruff SK (2013). Estrogen receptor beta ligand therapy activates PI3 K/Akt/mTOR signaling in oligodendrocytes and promotes remyelination in a mouse model of multiple sclerosis. Neurobiol Dis.

[17] Narayanan SP, Flores AI, Wang F, Macklin WB (2009). Akt signals through the mammalian target of rapamycin pathway to regulate CNS myelination. J Neurosci.

[18] Tyler WA, Gangoli N, Gokina P, Kim HA, Covey M, Levison SW, Wood TL (2009). Activation of the mammalian target of rapamycin (mTOR) is essential for oligodendrocyte differentiation. J Neurosci.

[19] Zhang Y, Chen K, Sloan SA, Bennett ML, Scholze AR, O’Keeffe S, Phatnani HP, Guarnieri P, Caneda C, Ruderisch N (2014). An RNA-sequencing transcriptome and splicing database of glia, neurons, and vascular cells of the cerebral cortex. J Neurosci.

[20] Butt AM, Fern RF, Matute C (2014). Neurotransmitter signaling in white matter. Glia.

[21] Khorchid A, Cui Q, Molina-Holgado E, Almazan G (2002). Developmental regulation of alpha 1A-adrenoceptor function in rat brain oligodendrocyte cultures. Neuropharmacology.

[22] Gupta MK, Papay RS, Jurgens CW, Gaivin RJ, Shi T, Doze VA, Perez DM (2009). alpha1-Adrenergic receptors regulate neurogenesis and gliogenesis. Mol Pharmacol.

[23] Imamura O, Arai M, Dateki M, Ogata T, Uchida R, Tomoda H, Takishima K (2015). Nicotinic acetylcholine receptors mediate donepezil-induced oligodendrocyte differentiation. J Neurochem.

[24] Karadottir R, Cavelier P, Bergersen LH, Attwell D (2005). NMDA receptors are expressed in oligodendrocytes and activated in ischaemia. Nature.

[25] Sung DJ, Kim JG, Won KJ, Kim B, Shin HC, Park JY, Bae YM (2012). Blockade of K+ and Ca2+ channels by azole antifungal agents in neonatal rat ventricular myocytes. Biol Pharm Bull.

[26] Lee PR, Fields RD (2009). Regulation of myelin genes implicated in psychiatric disorders by functional activity in axons. Front Neuroanat.

[27] Bebo BF, Dehghani B, Foster S, Kurniawan A, Lopez FJ, Sherman LS (2009). Treatment with selective estrogen receptor modulators regulates myelin specific T-cells and suppresses experimental autoimmune encephalomyelitis. Glia.

[28] Elloso MM, Phiel K, Henderson RA, Harris HA, Adelman SJ (2005). Suppression of experimental autoimmune encephalomyelitis using estrogen receptor-selective ligands. J Endocrinol.

[29] Jung-Testas I, Renoir M, Bugnard H, Greene GL, Baulieu EE (1992). Demonstration of steroid hormone receptors and steroid action in primary cultures of rat glial cells. J Steroid Biochem Mol Biol.

[30] Morales LB, Loo KK, Liu HB, Peterson C, Tiwari-Woodruff S, Voskuhl RR (2006). Treatment with an estrogen receptor alpha ligand is neuroprotective in experimental autoimmune encephalomyelitis. J Neurosci.

[31] Li R, Xu W, Chen Y, Qiu W, Shu Y, Wu A, Dai Y, Bao J, Lu Z, Hu X (2014). Raloxifene suppresses experimental autoimmune encephalomyelitis and NF-kappaB-dependent CCL20 expression in reactive astrocytes. PLoS ONE.

[32] Sicotte NL, Liva SM, Klutch R, Pfeiffer P, Bouvier S, Odesa S, Wu TC, Voskuhl RR (2002). Treatment of multiple sclerosis with the pregnancy hormone estriol. Ann Neurol.

[33] Amato MP (2005). Donepezil for memory impairment in multiple sclerosis. Lancet Neurol.

[34] Amato MP, Portaccio E (2011). Is there a future for donepezil therapy in the treatment of multiple sclerosis-related cognitive impairment?. Expert Rev Neurother.

[35] O’Carroll CB, Woodruff BK, Locke DE, Hoffman-Snyder CR, Wellik KE, Thaera GM, Demaerschalk BM, Wingerchuk DM (2012). Is donepezil effective for multiple sclerosis-related cognitive dysfunction?. A critically appraised topic. Neurologist.

[36] Cole R, de Vellis J (2001). Preparation of astrocyte, oligodendrocyte, and microglia cultures from primary rat cerebral cultures.

[37] Passaquin AC, Schreier WA, de Vellis J (1994). Gene expression in astrocytes is affected by subculture. Int J Dev Neurosci.

