# Supplementary material for: A high throughput drug screening assay to identify compounds that promote oligodendrocyte differentiation using acutely dissociated and purified oligodendrocyte precursor cells
Source: BMC Res Notes. 2016 Sep 5;9(1):419. doi: 10.1186/s13104-016-2220-2 (PMC5011342; doi:10.1186/s13104-016-2220-2)
Supplement: Supplementary file 1 — 10.1186/s13104-016-2220-2 Confirmation of OL differentiation hit compounds with multiple markers. [file 13104_2016_2220_MOESM1_ESM.pdf]

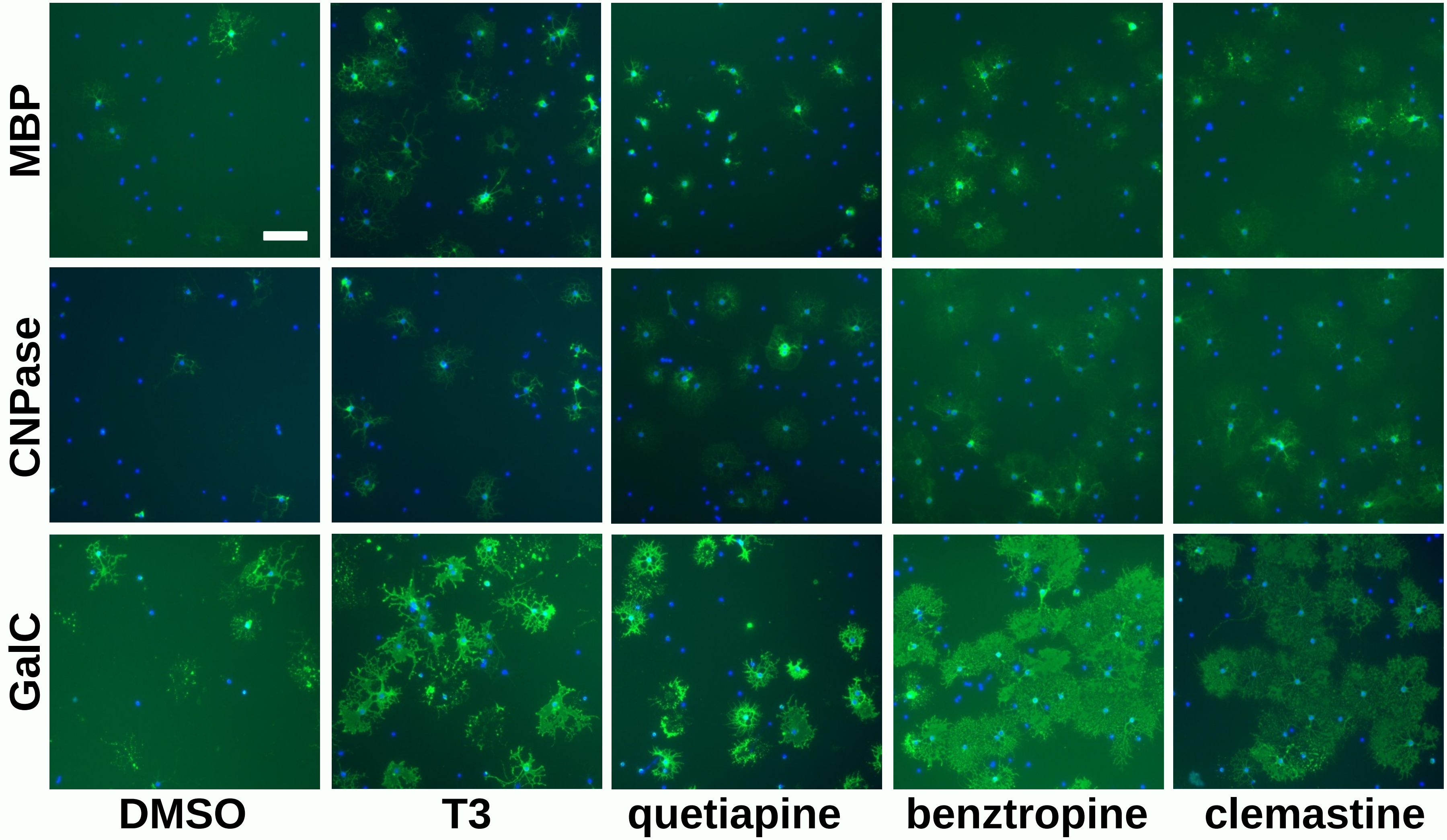

Figure S1A: Confirmation of OL differentiation hit compounds with multiple markers. Acutely purified OPCs were treated with test compounds for 4 days, fixed and immunostained with the differentiated OL marker antibodies to MBP, CNPase and GalC. Images shown are representatives of each of the classes in which the compounds were grouped in Table 1. Images are taken from two preparations and processed for presentation using identical parameters. The concentrations of the test compounds were DMSO-0.1%, T3-40 ng/ml, quetiapine-1 µM, benztropine-750 nM, clemastine-400 nM. Bar = 200 µM. Images are not shown for all compounds, but a representative from each drug class is shown.

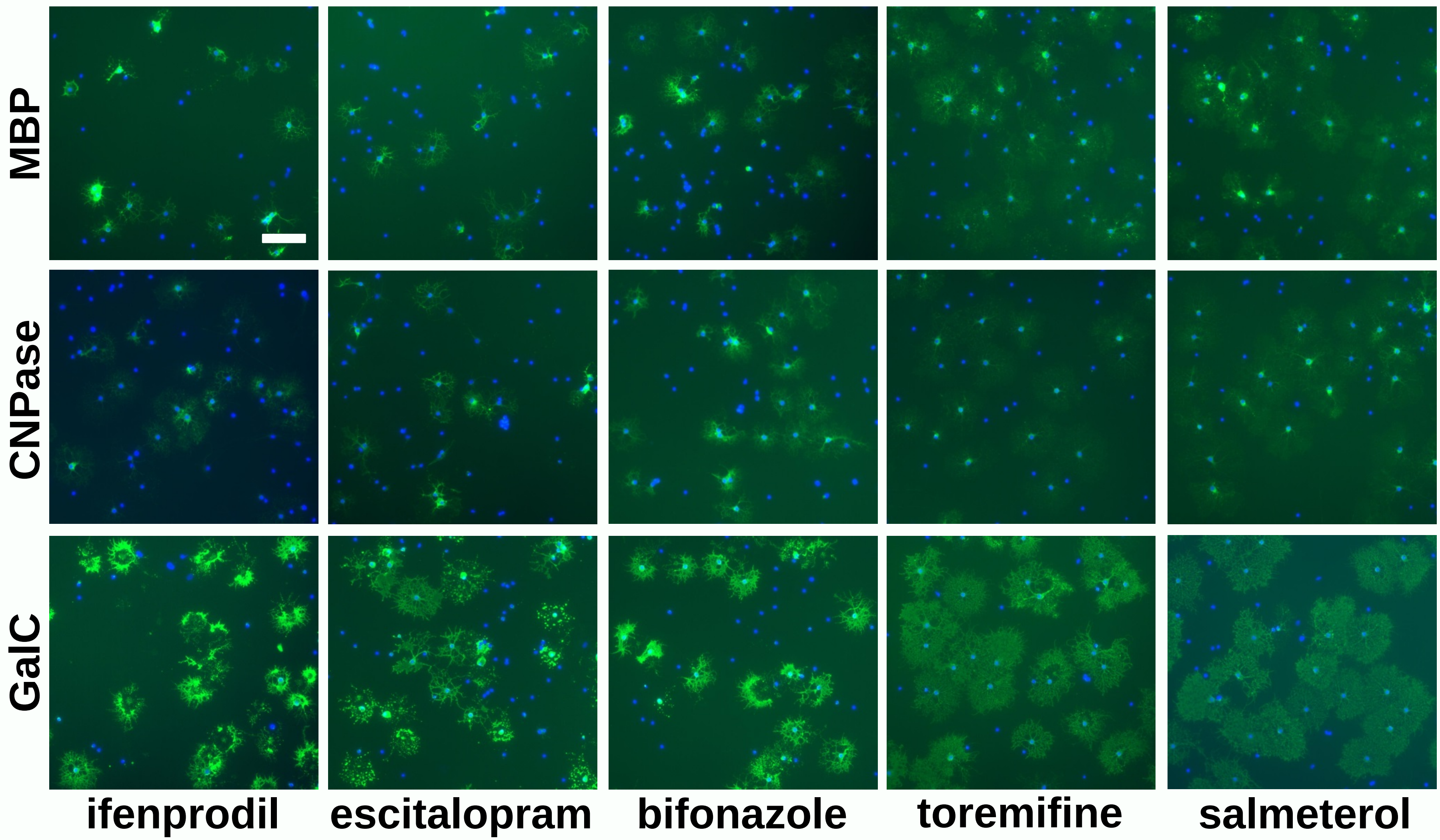

Figure S1B: Confirmation of OL differentiation hit compounds with multiple markers. Acutely purified OPCs were treated with test compounds for 4 days, fixed and immunostained with the differentiated OL marker antibodies to MBP, CNPase and GalC. Images shown are representatives of each of the classes in which the compounds were grouped in Table 1. Images are taken from two preparations and processed for presentation using identical parameters. The concentrations of the test compounds were ifenprodil-500 nM, escitalopram-3 µM, bifonazole-1 µM, toremifine- 200 nM, salmeterol-500 nM. Bar = 200 µM.
