# Supplementary material for: A high throughput drug screening assay to identify compounds that promote oligodendrocyte differentiation using acutely dissociated and purified oligodendrocyte precursor cells
Source: BMC Res Notes. 2016 Sep 5;9(1):419. doi: 10.1186/s13104-016-2220-2 (PMC5011342; doi:10.1186/s13104-016-2220-2)
Supplement: Supplementary file 4 — 10.1186/s13104-016-2220-2 Antibodies. [file 13104_2016_2220_MOESM4_ESM.pdf]

Table S1. Antibodies

Antibody	Source	Host	Dilution	Cell Marker
Myelin basic protein (MBP)	Abcam ab7349	Rat	1:500	Oligodendrocyte
Olig2	Millipore AB9610	Rabbit	1:1500	OPCs and Oligodendrocyte
CNP	Millipore MAB326	Mouse	1:1000	Oligodendrocyte
GalC	Hybridoma supernatant*	Mouse	1:25	Oligodendrocyte
Anti-rat Alexa Fluor 488	Molecular Probes	Goat	1:1000	Secondary antibody
Anti-rabbit Alexa Fluor 594	Molecular Probes	Goat	1:1000	Secondary antibody
Anti-mouse Alexa Fluor 594	Molecular Probes	Goat	1:1000	Secondary antibody

*Ranscht et al., 1982
